# Anatomy, Pathophysiology, Molecular Mechanisms, and Clinical Management of Erectile Dysfunction in Patients Affected by Coronary Artery Disease: A Review

**DOI:** 10.3390/biomedicines9040432

**Published:** 2021-04-16

**Authors:** Giuseppe Sangiorgi, Alberto Cereda, Daniela Benedetto, Michela Bonanni, Gaetano Chiricolo, Linda Cota, Eugenio Martuscelli, Francesco Greco

**Affiliations:** 1 Department of Biomedicine and Prevention, University of Tor Vergata, 00133 Rome, Italy; dania.benedetto@gmail.com (D.B.); michelabonanni91@gmail.com (M.B.); nucciochiricolo@gmail.com (G.C.); lindacota77@gmail.com (L.C.); eugeniomartuscelli@gmail.com (E.M.); 2 Cardiac Catheterization Laboratory, Department of Cardiology, San Gaudenzio Hospital, 28100 Novara, Italy; tskcer@hotmail.it; 3 Urologic Clinic, Centro Salute Uomo, 24125 Bergamo, Italy; francesco_greco@ymail.com

**Keywords:** erectile dysfunction, peripheral atherosclerotic disease, smooth muscle cells, Nitrix Oxide

## Abstract

Erectile dysfunction (ED) has been defined as the inability to attain or maintain penile erection sufficient for successful sexual intercourse. ED carries a notable influence on life quality, with significant implications for family and social relationships. Because atherosclerosis of penile arteries represents one of the most frequent ED causes, patients presenting with it should always be investigated for potential coexistent coronary or peripheral disease. Up to 75% of ED patients have a stenosis of the iliac-pudendal-penile arteries, supplying the male genital organ’s perfusion. Recently, pathophysiology and molecular basis of male erection have been elucidated, giving the ground to pharmacological and mechanical revascularization treatment of this condition. This review will focus on the normal anatomy and physiology of erection, the pathophysiology of ED, the relation between ED and cardiovascular diseases, and, lastly, on the molecular basis of erectile dysfunction.

## 1. Introduction

The Fourth International Consultation on Sexual Medicine has defined erectile dysfunction (ED) as the consistent or recurrent inability to attain and maintain penile erection sufficient for sexual satisfaction [1] ED is classified as organic, psychological, or resulting from several simultaneous (mixed) factors, the most frequent form. Today, it is still problematic to accurately estimate the impact and the incidence of ED because of social, ethical, cultural, and religious reasons. Moreover, many men are convinced that sexual impairment is an inevitable feature of late age [2,3] leading to reduced and delayed medical advice. 

The global mean ED prevalence ranges from 14% to 48%, with higher rates in the US and South East Asia than European rates [1,4]. In the U.S., at least 12 million men between 40 and 79 years of age have ED. In contrast, Italy has reported a prevalence of ED (complete and incomplete) of 12.8% and a significant incidence of age-related ED (2% between 18 and 30 years and 48% over 70 years) [5]. Based on the Massachusetts Male Aging Study (MMAS) data, over a population range between 40 and 70 years, ED increased with age from 5.1% to 15% and from 17% to 34% for complete and moderate ED, respectively; mild ED remained stable at about 17% over the years. Furthermore, the prevalence of ED worldwide will be estimated to reach 322 million men by 2025 [6]. Perhaps, this percentage is significantly underestimated.

Increasing evidence suggested an association between ED and cardiovascular diseases (CVD) [7] with an increased prevalence of ED in cardiovascular patients and an increased prevalence of CVD in patients with ED [8]. However, among clinical manifestations of atherosclerotic disease, ED usually proceeds by approximately five years the onset of coronary diseases, such as coronary disease, begins five years early the onset of carotid and peripheral disease with claudication [9].

Performance anxiety and relationship issues are commonly recognized psychological causes of ED. Still, its prevalence is also related to several age-independent comorbidities, such as congestive heart diseases, atherosclerosis, blood hypertension, and other vascular disorders, psychiatric disorders (depression), endocrine disorders (diabetes, reduction of testosterone), neurological disorders, and concomitant other genitourinary disease related to surgery [10].

This review will summarize the mechanism of men’s erection, focusing on pathophysiology and molecular mechanisms of erectile dysfunction, with a glimpse of the clinical linkage between cardiovascular disease and ED.

## 2. Anatomy of Erection

Erection is a neurovascular event that consists of a vascular phase that is the consequence of a balance between arterial inflow and venous outflow. The penis comprises three cylindrical structures: the paired corpora cavernosa (which represent the dorsal and lateral portion) and the corpus spongiosum, which is contained the urethra (which represent the ventral portion). All these structures are covered by a loose subcutaneous layer and skin. The tunica albuginea is a structure made up of bundles of collagen fibers and is bi-layered. The outer-layer bundles are oriented longitudinally from the glans penis to the proximal crura, while the inner-layer bundles are oriented circularly and house the corpora cavernosa. These two main layers are connected by oblique-oriented fibers. Emissary veins are contained between these two layers and are compressed during erection by the outer one. The thickness of the albuginea varies among the individuals and the different locations (it is more significant at the ventrolateral level). On average, however, it measures between 2–3 mm when the penis is flaccid and reduces to 0.5 mm during erection, providing rigidity to the corpora cavernosa and participating in the veno-occlusive mechanism.

The penile corpora cavernosa can increase their volume during sexual arousal by changing the contraction of cavernosal smooth muscle cells (SMCs). Central triggers and peripheral transmitters systems control the balance between contraction and relaxation. In particular, the trabecular SMCs represent approximately 40–50% of the cavernosal tissue cross-sectional area [11,12]. The remaining cavernosal tissue is made by extracellular matrix, predominantly constituted by collagen fibers, types I, III, IV, and elastin [13,14], and by a minority part of collagen types V and XI. In this highly specialized anatomical structure, endothelial cells and neurons are critical for maintaining and regulating vascular smooth muscle cell (VSMC) tone. This complex architecture is supported by the expression of numerous growth factors, such as sonic hedgehog (Shh), which, in turn, triggers the expression of vascular endothelial growth factor (VEGF) and NOS in the penis [15].

Furthermore, from a pathophysiologic perspective, the corpora cavernosa are represented by a conglomeration of sinusoids, larger at the centre and smaller near the periphery. During the flaccid state, the blood tends to circulate centrifugally from the central to the peripheral sinusoids. Conversely, during the erection state, the blood rapidly flows to central and peripheral sinusoids [16]. The SMCs regulate the blood flow inside and outside the sinusoids. Even though the corpus spongiosum has a similar structure to the corpora cavernosa, it has no outer layer, guaranteeing lower pressure during erection. 

The primary source of penile blood derives from the internal iliac artery branch: the internal pudendal artery. However, there could be accessory arteries (branches of the external iliac, vesical, obturator, and femoral arteries). In some men, they could represent the main or the only blood supply to the corpora cavernosa [17]. The internal pudendal artery ends in the common penile artery with three branches represented by the dorsal, bulbourethral, and cavernous artery. The latter lies in the middle of the corporal bodies and gives rise to the helicine resistance arteries, which open directly into the lacunar spaces and feed the individual trabeculae. The blood from the penis is depleted by three veins, including superficial, intermediate, and deep [18]. The superficial veins primarily drain the skin of the penis, and they communicate with the deep dorsal veins. The intermediate veins mainly consist of the deep dorsal vein (which drains the cavernous tissue) and prostatic or crural venous plexus, representing the target for venous insufficiency surgical ligation or percutaneous embolization. 

**Figure 1:**
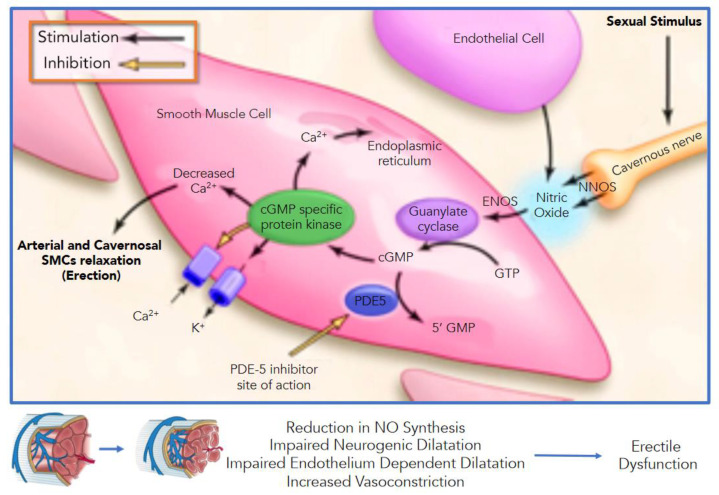
Pathways of penile erection: Dilation of cavernosal arteries and smooth muscle cells (SMCs) of cavernosal spaces is mediated by the nitric oxide (NO)–cyclic guanosine monophosphate (cGMP) pathway. NO is derived from nonadrenergic, noncholinergic nerves (nNOS, NOS type I), and the endothelium (eNOS or NOS type III) of cavernosal sinuses (both calcium^2+^/calmodulin-dependent isoforms). NO crosses plasma membranes to enter vascular smooth muscle cells (VSMCs) and promotes the synthesis and accumulation of cGMP, which induces a cascade reaction, leading to smooth muscle relaxation by reducing intracellular calcium level. NNOS = neuronal nitric oxide. ENOS = endothelial nitric oxide. See the text for details.

The innervation of the penis is both autonomic and somatic. Somatic innervation (responsible for sensation and muscle contraction) arises from the second to fourth sacral spinal cord segments. These nerves travel into the pudendal nerve to innervate the ischiocavernosus and the bulbocavernosus muscles responsible, respectively, for the rigid erection phase and the semen expulsion. Autonomic innervation is made of sympathetic nerves originating from the 11th thoracic to the 2nd lumbar spinal segments and travel to the sympathetic chain ganglia and parasympathetic nerves that arise from the second, third, and fourth sacral spinal cord segments (pelvic nerves). Some sympathetic fibers run through the lumbar splanchnic nerves to the superior hypogastric and the inferior mesenteric plexuses. The parasympathetic fibers’ pelvic nerves pass into the pelvic plexus, where they merge with the sympathetic nerves arising from the superior hypogastric plexus. Finally, the cavernous nerves, originating from the pelvic plexus, are responsible for penile innervation. Triggering of the pelvic plexus and the cavernous nerves leads to the erection, while detumescence is the consequence of sympathetic nerve stimulation (Figure 1).

A physiological penile erection requires normal vascular, neurological, and tissue responses. Penile erection is initiated after the central stimulus and integration of tactile, visual, olfactory, and imaginative triggers. Different observations demonstrate a network of afferents fibers from the genitals, spinal interneurons, and sympathetic, parasympathetic, and somatic nuclei, receiving stimulus from the periphery at the spinal cord level triggering reflexive erections. This network seems to receive also supraspinal information. At the level of the central nervous tissue, afferent data is processed in the forebrain and hypothalamus. In this latter area, the medial preoptic center, paraventricular nucleus, and anterior hypothalamic regions regulate erections and modulate autonomic events associated with sexual response. Different efferent pathways are present between the hypothalamus and sacral autonomic centers represented by the thoracolumbar and sacral autonomic nuclei. The erectile activity is finally coordinated by autonomic fibers network to the penis and somatic fibers pathways to the perineal striated muscles, responsible, in turn, for penis sensation. The cavernosal nerves originate from the autonomic system and are made of both sympathetic and parasympathetic fibers. These fibers travel along the prostate and enter the corpus spongiosum and the corpora cavernosa to regulate the blood flow during erection. The central regulation of penile erection involves many transmitters, but their specific function is unknown [19,20]. Nerve impulses trigger neurotransmitters’ release and relaxing factors from the endothelial cells, resulting in smooth muscle cells’ relaxation with a consequent increase in blood flow in the cavernous bodies arterioles. Simultaneously, the trabecular smooth muscle relaxation increases sinusoid compliance. This, in turn, increases the cavernous system’s rapid filling and volume enlargement. The vein plexuses are then compressed between the trabeculae and the tunica albuginea, resulting in almost complete occlusion of venous outflow. Thus, these events block the blood within the corpora cavernosa and raise the penis from a flaccid to an erectile state, with an intracavernosal pressure of approximately 100 mmHg. Detumescence results from neurotransmitter release cessation, followed by second messengers’ breakdown by phosphodiesterase’s or sympathetic discharge during ejaculation. The trabecular smooth muscle contraction decreases the volume of the corpora cavernosa, followed by re-opening of the veins and consequent blood out-drainage from the cavernous bodies and returning to a flaccid state.

In summary, to have a normal erection, several components are required: (a) functioning nervous system; (b) good arterial flow; (c) healthy corpora cavernosa; (d) the ability to block the venous blood spill.

## 3. Vascular Events of Erection 

Erection is a combination of sinusoid relaxation, arterial dilation, and venous compression. The cavernous smooth musculature and the SMCs of the arteriolar and arterial walls play a fundamental role in the erectile process: in the flaccid state, these smooth muscles are tonically contracted, avoiding the blood to flow correctly into the corpora. Alpha-adrenergic nerve fibers, both alpha1 and alpha2, and their receptors are located in the cavernous trabeculae and cavernous arteries. Post-junctional alpha1-adrenergic receptors trigger contraction, whereas prejunctional alpha2-adrenergic receptors activation downregulates neurotransmitters’ release (norepinephrine and nitrile oxide, NO) [21,22,23]. Norepinephrine is known to be the principal neurotransmitter involved in the flaccid state. Adrenergic nerves release norepinephrine to stimulate the adrenergic system: the bound with its receptor produces a contraction that involves Ca^2+^ entry through calcium channels and the activation of protein kinase C, tyrosine kinases, and Rho-kinase [20,21,24]. Endothelin-1 is produced by endothelium and acts as a potent vasoconstrictor, more than epinephrine: inducing long-lasting smooth muscle contractions, enhancing catecholamine-mediated constrictor effects on trabecular smooth muscle, and being considered a mediator for detumescence [25]. 

When arousal occurs, parasympathetic activity from the spinal cord’s sacral segments triggers the cavernous nerve terminals to release neurotransmitters. Sexual function is related to numerous neurotransmitters and neuropeptides, and the main ones are dopamine, oxytocin, nitrile oxide (NO), norepinephrine, serotonin (5-hydroxytryptamine), and prolactin. Acetylcholine is no more considered as the primary neurotransmitter. However, by presynaptic inhibition of adrenergic neurons and the release of NO by endothelial cells, it contributes indirectly to penile erection [26]. 

An intricate balance between the central and peripheral nervous system and the integrity of the penile vasculature determines the ability to pass from flaccid state to erection. The central nervous system’s response to sexual stimulation is mediated by the hypothalamus region through dopamine release, with the production of impulses through the spinal cord to stimulate the erection process. Furthermore, the same process can be achieved through direct tactile stimulation of the penis through the production of erectile stimuli to the sacral cord by excitatory sensory neurons at the level of the S2–S4 region. Therefore, efferent neurons emerge through the neural sacral foramen forming synapses with postganglionic fibers and non-adrenergic noncholinergic (NANC) fibers in the hypogastric plexus. These fibers run through the cavernous nerves at the level of the corpora cavernosa and generate reflexes (central or local starting impulses) that cause the release of NO. The vascular endothelium further releases NO molecule in response to parasympathetic stimulation and acetylcholine release, linked to increased wall stress due to increased blood flow in the cavernous sinusoids.

Today, NO released from noncholinergic fibers and the endothelium is considered the predominant neurotransmitter responsible for penile erection. NO is produced by the enzyme NO synthase (NOS). Three different subtypes of NOS have been identified and differentiated by the source where they were isolated: nNOS (n = neuronal), iNOS (I = immunoactivated macrophage cells line), and eNOS (e = vascular endothelium). All NOS subtypes produce NO, but they play a different biological role in various tissues. nNOs and eNOS are calcium-dependent forms. The former subtype regulates neurotransmission while the latter regulates blood flow. Conversely, iNOS is calcium-independent, and it is mainly involved in carcinogenesis. 

NO spreads at the cellular level and activates the guanylate cyclase (Figure 1), increasing the production of cGMP (cyclic guanosine monophosphate), which relaxes the cavernous smooth muscle [27,28,29,30] Through a protein-kinases mediated cascade (phosphatidylinositol-3-kinase/protein kinase B (Akt)), hyperpolarization of the cell membrane and intracellular calcium sequestration occurs, leading to the release of smooth muscle cells of the corpora cavernosa and arterial vasodilation. The cavernous sine waves’ swelling simultaneously causes compression of the venous plexus in the tunica albuginea, resulting in venous outflow obstruction and maintaining the erectile phase. The phosphodiesterase-5 compounds are the mediators of the return to the state of penile detumescence through cGMP hydrolysis. The pro-erectile parasympathetic mechanism is offset by adrenergic sympathetic fibers that run in the cavernous nerves. The release of norepinephrine from sympathetic neurons stimulates and maintains the flaccidity by releasing alpha-1 G-protein receptors on SMCs of the cavernous sinusoids, which activates calcium ions decrease in the intra-cytoplasmic reticulum and subsequent relaxation of smooth muscle cells themselves.

Thus, NO-induced smooth muscle relaxation allows a series of events to occur: first, dilation of the arterioles, increasing the blood inflow; second, dilation of sinusoids, entrapping the incoming blood; third, reduced venous outflow by the compression of the sub-tunical venous plexuses; and, finally, elongation and shrinking of the tunica, contraction of ischiocavernosus muscle occluding the emissary veins and increase of the intra-cavernous pressure to a level higher than systolic pressure (the full erection phase). During sexual stimulation, reflex contractions of the ischiocavernosus muscles (rigid-erection phase) can increase additional pressure. Corpus spongiosum and glans penis act differ from corpora cavernosa: spongiosum and glans essentially work as a large arteriovenous shunt during the erection phase. In this phase, the arterial flow increases similarly; however, the corpus spongiosum and glans’ flow pressure represent only 30–50% of all corpora cavernosa. This is due to the tunica albuginea (thin at the corpus spongiosum and practically absent on the glans), guarantying weaker venous occlusion. In the full-erection phase, partial compression of the deep dorsal and circumflex veins contributes to glans tumescence. In the rigid-erection stage, the ischiocavernosus and bulbocavernosus muscles compress the spongiosum and penile veins, contributing to additional engorgement and increased pressure in the glans spongiosum. After ejaculation, the smooth muscles contract gradually, reducing intra-cavernous pressure, leading to venous relaxation and, consequently, blood outflow, supported by the breakdown of cGMP by PDEs (phosphodiesterases), the cessation of NO release, and the sympathetic discharge (responsible for ejaculation).

## 4. Molecular Basis of Erectile Dysfunction

Several studies have shown that within the central nervous system, NO can modulate sexual arousal and erection [31,32,33]. NO may act in several brain regions, and the paraventricular nucleus [34], and an increase in NO production in this area has been demonstrated in experimental animal studies during copulation [35]. NO may also mediate by Adrenocorticotropic hormone/a-Melanocyte Stimulating Hormone (ACTH/a-MSH) and 5-Hydroxytryptamine2C (5-HT2C) agonists’ actions, which, in turn, may trigger erections when injected into the ventricular brain system [32]. Furthermore, the inhibitory effect of NOS inhibitors is not observed when these compounds are injected together with L-arginine, the substrate for NO [36]. In the penis, the two principal sources of NO are the nonadrenergic, non-cholinergic nerves and the endothelium of penile arteries and cavernous bodies [27,37]. 

**Figure 2:**
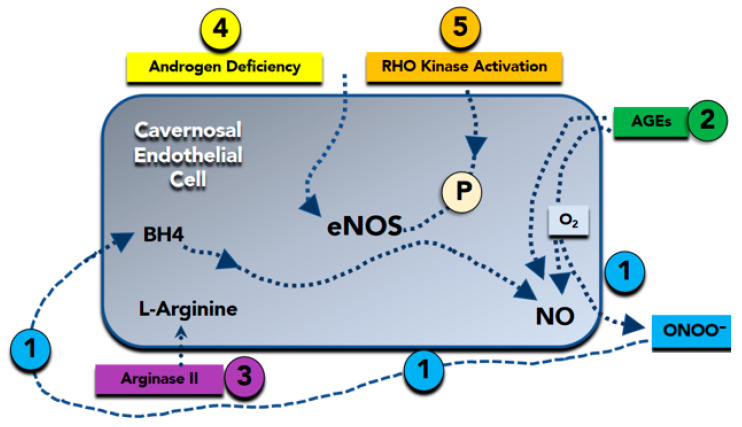
Molecular basis of nitric oxide (NO) reduction in erectile dysfunction. (Bullet 1) Superoxide (O_2_^−^) inactivates NO to form peroxynitrite (ONOO^−^). Peroxynitrite attacks and destroys tetrahydrobiopterin (BH4), a critical cofactor in NO synthesis. (Bullet 2) Advanced glycation end products (AGEs) promote superoxide formation, as well as directly inactivate NO. (Bullet 3) Arginase II degrades L-arginine, a substrate required for NO synthesis. (Bullet 4) Androgen deficiency reduces the expression of the endothelial isoform of NO synthase (eNOS). (Bullet 5) Rho-kinase reduces expression and phosphorylation of eNOS, which is required for its full activation. P = eNOS phosphorylation.

The primary electromechanical mechanism of contraction in VSMCs involves depolarization and opening of voltage-gated L-type Ca^2+^ electromechanical channels, which allows the influx of extracellular Ca within the cell. The opening of the Ca-dependent potassium channels on the membrane leads to potassium outflux and hyperpolarization. Finally, the cytosolic Ca + + depletion causes cavernosal SMC relaxation leading to increased blood inflow through the helical arteries, sinusoidal filling and cavernosal dilation. At the same time, VSMC relaxation is related to the opening of K^+^ channels (Figure 2). Moreover, membrane potential changes due to increased K^+^ efflux inactivate L-type Ca^2+^ channels inhibit Ca^2+^ influx. Thus, NO may also cause VSMC hyperpolarization. NO diffuses to SMCs, where it augments the formation of cGMP (nitric oxide-cyclic guanosine monophosphate (cGMP)), which acts as a second messenger [27,35,36,37,38]. Then, cGMP that accumulates in SMCs is broken down by phosphodiesterase (PDE) enzymes [28,39], with PDE5 (phosphodiesterase-5) being the predominant isoform in the corpus cavernosum [39] (Figure 2). The same NO-cGMP pathway at the base of a healthy erectile function is the critical endothelium-derived pathway for vascular dilatation in the systemic and coronary circulation [23,40] which explains the tight pathogenic correlation between ED and coronary artery disease. Furthermore, NO mediates many of the endothelium’s antiatherogenic functions by blocking the expression of proinflammatory cytokines, chemokines, and leukocyte adhesion molecules [41]. 

Therefore, loss of the biologic activity of endothelium-derived NO is coupled by other alterations in endothelial phenotype that further increase the propensity for systemic vasoconstriction, inflammation, and cellular proliferation [42]. Furthermore, a reduction in NO availability due to impaired endothelial eNOS activity (Figure 2) and the subsequent increase in vasoconstriction causes a decrease in blood flow and oxygen supply, favoring free oxygen radical production. This inflammatory substrate favors, in turn, cavernous bodies fibrosis and progressive loss of erectile function [19,20,21,24]. 

## 5. Erectile Dysfunction and Cardiovascular Diseases

Among the different pathogenic mechanisms of ED (Table 1), vascular etiology is the most common cause [43]. As a known symptom of atherosclerotic lesions, ED shares the same modifiable risk factors with coronary artery disease and peripheral artery disease, including hypertension, diabetes, dyslipidemia, cigarette smoking, obesity, and metabolic syndrome sedentary behavior. 

ED is an independent risk factor for future cardiovascular events, being a potentially useful marker for cardiovascular disease [44,45,46,47]. ED commonly accompanies silent heart disease [42,43,44] with an average time interval between the onset of ED and coronary heart disease by 2 to 5 years (class Ia) [48,49]. A recent metanalysis by Osondu and co-authors [50] confirms an association between ED and subclinical cardiovascular diseases identified by different variables, such as endothelial dysfunction, with impaired flow-mediated dilatation, carotid intimal medial thickness, coronary artery calcification, ankle-brachial index, underscoring the importance of aggressive cardiovascular disease risk assessment and management in patients affected as the first onset by ED. Furthermore, a multicenter prospect cohort study [51] in 1757 participants during a 3.8-year follow-up (interquartile range, 3.5–4.2) was recently published. Eight hundred and seventy-seven (45.8%) participants reported ED symptoms. Patients affected by ED were more likely to have diabetes mellitus and positive family history of coronary heart disease (CHD). Patients were also more likely to utilize β-blocker, antihypertensive, lipid-lowering, and antidepressant drugs. Over follow-up, a total of 40 CHD and 75 cardiovascular disease (CVD) hard events occurred in this cohort. In particular, a significantly greater proportion of patients affected by ED experienced hard events compared to those without ED (CVD hard events: 6.3% versus 2.6%, *p* < 0.001; CHD hard events: 3.4% versus 1.4%, *p* < 0.001). In the unadjusted Cox models, ED was a significant predictor of both hard CHD (hazard ratio, 2.5; 95% confidence interval [CI], 1.3–4.8), and CVD (hazard ratio, 2.6; 95% CI, 1.6–4.1) events. In the fully adjusted models, ED remained a significant predictor of hard CVD events (hazard ratio, 1.9; 95% CI, 1.1–3.4), whereas hard CHD events not.

**Figure 3:**
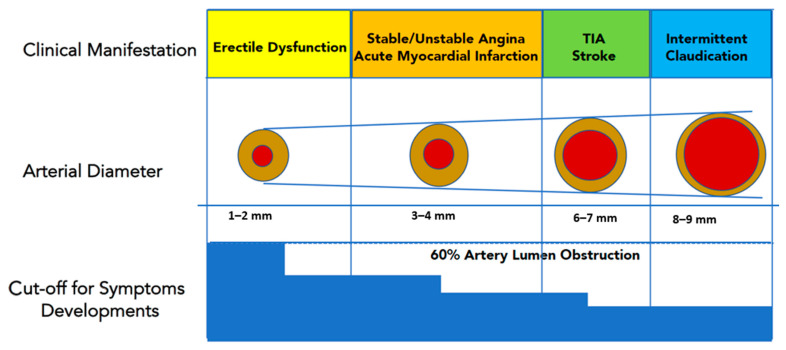
Luminal narrowing due to atherosclerotic burden will manifest clinically earlier in penile arteries (smaller caliper) than in coronary, carotid, or iliac district (larger caliper). This explains why ED represents the “tip of the iceberg” of systemic atherosclerotic disease, and it is usually the first “alert sign” to manifest before a major cardiovascular event (Modified from Reference [9]). TIA, transient ischaemic attack.

Several explanations should be taken into account why ED is a precursor of CVD events. In this setting, Montorsi and co-authors [52] hypothesized that the smaller sized penile arteries (1–2 mm) would suffer earlier from atherosclerotic plaque burden, leading to arterial obstruction and flow compromise. Conversely, larger coronary (3–4 mm) or carotid arteries (5–7 mm) are affected later in the patient’s life span. Therefore, ED would represent early clinical evidence of a diffuse, systemic vascular disease, being “the tip of the iceberg” of preclinical cardiovascular disorders (Figure 3). Another explanation [53] is related to the increased arterial stiffness in the elderly, which can increase systolic blood pressure while decreasing diastolic blood pressure. The large-artery stiffness and subsequent systolic hypertension may force the pressure waves farther into smaller arteries leading to pudendal and penile arteries atherosclerosis. Abnormalities in the endothelial nitric oxide synthase (eNOS) production are also involved in endothelial dysfunction, which leads to ED and accelerated atherosclerosis. Indeed, ED has been associated with endothelial dysfunction of conduit vessels, increased coronary artery calcification, and silent angina independent of traditional cardiovascular risk factors [53]. Furthermore, a chronic hypoxemic stimulus is an independent risk factor for the development of ED; this occurs, for example, in obstructive sleep apnea syndrome and chronic lung disease. Hypoxia determines an increase in vasomotor tone and causes the stimulus for vascular growth factors production, inhibiting the endothelium-dependent relaxation, and favoring corporal arterioles vasoconstriction. 

Traditionally, major traditional cardiovascular risk factors, such as diabetes, hypercholesterolemia, hypertension, and cigarette smoking, promote endothelial dysfunction and, ultimately, vasculogenic ED [44,47,54,55]. Diabetic ED (DED) is associated to an insufficient response to NANC nerve stimulus and an inability to vasodilate small arterioles of the penis. This results from reduced production of eNOS related to endothelial dysfunction and atherosclerotic disease [19]. Epidemiologic data report that up to 75% of diabetic patients have a lifetime risk of developing ED [10] and ED in diabetics is more common than retinopathy or nephropathy [56]. Moreover, the clinical picture of ED is accelerated in diabetic patients: its onset occurs at an earlier age, presenting within ten years of the diabetic onset in more than 50% of patients with any type of diabetes [57]. Histopathological analysis of cavernous bodies specimens from diabetic men with ED demonstrated ultrastructural changes in the cavernous arteries, cavernous smooth muscle, and impaired endothelium-dependent relaxation of the corporeal SMCs [19]. In 12% of type 1 diabetic men, ED was the first symptom of diabetes [58]. The presence of ED in diabetic patients could be a significant precursor of cardiovascular disease. Gazzaruso et al. [59] found a higher prevalence of ED in diabetic patients with silent CHD than those without any evidence of myocardial ischemia. Moreover, ED was associated with higher major cardiovascular morbidity and mortality in diabetic patients with silent CHD. 

The prevalence of hypertension in the ED population is higher than in people without ED [60] and vice versa, a high prevalence of ED is generally observed in hypertensive patient populations [61]. Both antihypertensive drugs and hypertension alone can deteriorate the erectile function. High blood pressure is characterized by increased peripheral sympathetic activity, which maintains an elevated vasoconstrictor tone and decreases the endothelium-dependent vasodilation in arteries leading to consequent alterations in vessel architecture and diminished dilatory capacity; moreover, vascular remodeling could also occur at the corporal level, progressing to ED by altering mechanical properties of erectile tissue [61]. ED is usually associated with longer duration and more severe hypertension. In hypertensive rats, the impairment of cavernosal endothelium-dependent and NO donor-induced relaxations also occurred before systemic vascular alterations are manifested [62], suggesting that erectile tissue is an early end-organ target for developing endothelial dysfunction in this patients’ cohort. 

The association between ED and CVD in patients with preexisting cardiac conditions is complex and requires the interaction of urologists and cardiologists. In patients affected by ED, baseline investigations should include the assessment of ED using validated questionnaires, such as the International Index of Erectile Function in order to assess ED severity. Because episodic sexual activity could trigger acute cardiac events in specific CVD patients, exercise assessment is a critical step in managing ED. Evaluation should be done before a patient can be counseled about the safety of sexual intercourse and the use of pro-erectile drugs. The stress of sexual performance on the heart corresponds to a medium level of physical activity, as to complete 4 min of the standard Bruce treadmill test, up to 4–5 Metabolic Equivalents (METs). In general, if a heart patient can achieve this during exercise symptom-free level, should be able to have sex without cardiovascular problems. However, patients must be stratified by their likelihood of CVD events or their mortality both during and immediately after sexual activity.

Low-risk patients can safely perform sexual activity and should receive ED treatment. In this group are included men that were successfully revascularized, or men with asymptomatic controlled hypertension, mild valvular disease, and class I and II heart failure according to the New York Heart Association (NYHA) classification. High risk category indicated men with unstable angina, uncontrolled hypertension, NYHA class IV heart failure, myocardial infarction within two weeks without intervention, high-risk arrhythmia, symptomatic hypertrophic cardiomyopathy, and moderate to severe valve disease. These men should defer sexual activity until the cardiac condition has been stabilized, receive intensive risk-factor correction. Men not included in these two groups are considered as patients with indeterminate risk. Those patients should be reassessed using the stress test and, in turn, be reassigned to a low- or high-risk category [63].

Phosphodiesterase-5 Inhibitors in Patients affected by Coronary Artery Disease PDE5i have shown to effectively improve erectile function when assumed on demand and are now considered first-line pharmacotherapy for treatment of ED. PDE5i have an excellent safety profile and can be administered to CVD patients. The most widely prescribed PDE5i approved for the treatment of ED are Sildenafil citrate (Sildenafil), Tadalafil, and Vardenafil that have proven efficacy in treating erectile dysfunction and also are currently prescribed on pulmonary arterial hypertension (PAH). PDE5i shown some differences in their biochemical properties, pharmacokinetic profiles, and clinical performance. In particular, Tadalafil absorption does not seem to be influenced by the intake of fatty meals or alcohol; the peak of serum concentration is reached about 2 h after the dose instead of 1 h with the other two PDE5i, moreover half-life has a duration of 17.5 h compared with 3.7 h for sildenafil. Furthermore, Tadalafil administration improves erectile function up to 36 h post-dose. The theoretical impact of these pharmacokinetic properties is that spontaneous sexual activity can be more easily restored by chronically administering this drug. On the other hand, the prolonged half-life results in greater long-term adverse effects (such as headache) than other PDE5i. However, there is no consensus on which drug is most recommended for ED treatment. Patient’s choice and physician’s judgement must be considered when prescribing a PDE5i.

**Figure 4:**
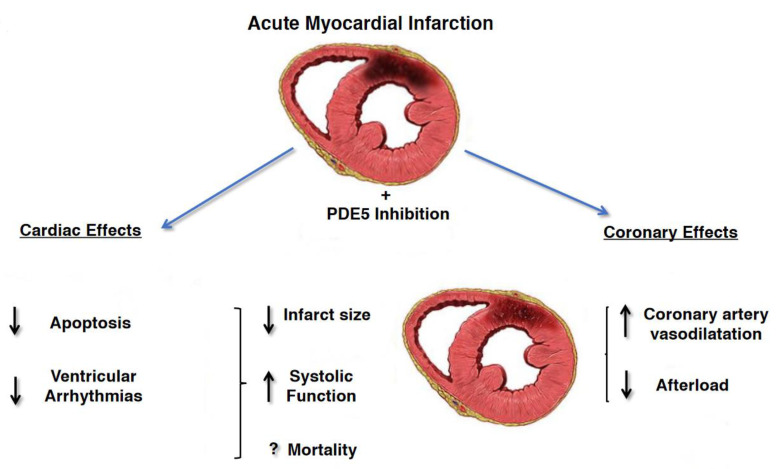
Cardioprotective effects of PDE5i in patients affected by acute myocardial infarction. Reduction of infarct size related to decrease myocardial cells’ apoptosis, decrease in afterload due to increase vasodilatation and increase in systolic function has been demonstrated (Modified from Ref. [70]).

The beneficial effects of PDE5i on the cardiovascular system are supported by numerous animal and human studies showing sustained improvement in hemodynamics parameters including arterial stiffness, flow-mediated dilation, and peak systolic velocity, even after discontinuation. These findings may be due to the positive effects of PDE5i on endothelial function and in particular on vasodilation, thrombosis and inflammation. In fact, PDE5i improve erectile function by increasing the availability of nitric oxide in the penis and its vascular system, resulting in vasodilation and increased blood flow. PDE5i could benefit cardiovascular disease because phosphodiesterase-5 is also found in other parts of the body, including the pulmonary and systemic vascular systems and in hypertrophic myocardium. PDE5i are in fact used in primary pulmonary arterial hypertension with reversible pulmonary arterial resistance. In addition, PDE5i appear to protect the myocardium through complex pathways involving nitric oxide, cyclic guanosine monophosphate, protein kinase G, extracellular signal-regulated kinase, B-cell lymphoma protein 2, and Rho kinase inhibition. In animal models of acute myocardial infarction, PDE5i consistently reduced the size of the infarct indicating cardio-protection. PDE5i also promote reverse remodeling and reduce myocardial apoptosis, fibrosis and hypertrophy [49,64,65]. Those cardiovascular beneficial effects were stronger in patients with prior myocardial infarction (MI) and were associated with reduced incidence of new MI, raising the possibility that PDE5is could prevent both complications post-MI and future cardiovascular events [66] (Figure 4) In particular, in a Swedish nationwide cohort study, 43.145 men <80 years of age without prior MI, or cardiac revascularization, hospitalized for MI during 2007–2013 were evaluated for the risk of death, MI, cardiac revascularization or heart failure after treatment with PDE5i or alprostadil [67]. Men with, compared with those without treatment for ED, had a 33% lower mortality (adjusted Heart Ratio (HR) 0.67 (95% CI 0.55 to −0.81)), and 40% lower risk of hospitalization for heart failure (HR 0.60 (95% CI 0.44 to 0.82)). There was no association between treatment with alprostadil and mortality. The adjusted risk of death in men with 1, 2–5 and >5 dispensed prescriptions of phosphodiesterase-5 inhibitors was reduced by 34% (HR 0.66 (95% CI 0.38 to 1.15)), 53% (HR 0.47 (95% CI 0.26 to 0.87)), and 81% (HR 0.19 (95% CI 0.08 to 0.45)), respectively, when compared with alprostadil treatment, suggesting a dose-dependent effect of PDE5i. Another study, enrolled 5956 pts, aged 40–89 years, with a prior history of type 2 diabetes with high attendant cardiovascular risk [68] to describe the potential cardioprotective action of on-demand PDE5i administration in overall mortality. Diabetic pts treated with PDE5i was associated with a significant lower rates of incident MI (incidence rate ratio 0.49 to 0.80), *p* < 0.0001) with lower all-cause mortality and a lower proportion of death (25.7% vs. 40.1% deaths; *p* = 0.001) compared with non-users. This lower mortality risk in those taking a PDE5i persisted after adjustment for known risk modifiers including previous stroke, previous MI, age, estimated glomerular filtration rate (eGFR), CVD, hypertension and use of cardioprotective agents, such as β-blockers and statins. In addition, in a subgroup analysis of patients with history of MI or an incident MI during the study period, PDE5i use was associated with significantly lower mortality risk. A recent nationwide observational cohort study [69] enrolled 18,542 men with stable CHD; 16,548 men were treated with PDE5i and 1994 pts were treated with alprostadil. The mean follow-up was 5.8 years, with 2261 deaths (14%) in the PDE5i group and 521 (26%) in the alprostadil group. Moreover, men with PDE5i treatment showed lower long-term risk of all-cause and cardiovascular mortality, MI, heart failure, and cardiac revascularization after adjustment for potential confounders, including marital status and length of education.

## 6. Guidelines for Therapeutic Management of Erectile Dysfunction 

The American Urological Association and the European Urology Association have recently published new guidelines on the management of erectile dysfunction [71,72], to offer a high-quality resource for assisting clinicians and patients in understanding the benefits and risks/burdens of the various management strategies for ED. The first management strategy consists of lifestyle modifications, including diet changes and increased physical activity, which improve overall health and may improve erectile function (*Moderate Recommendation; Evidence Level: Grade C*). The second recommendation is that men affected ED should be informed regarding the treatment option of oral phosphodiesterase type 5 inhibitor (PDE5i), unless contraindicated. (*Strong Recommendation; Evidence Level: Grade B*). In addition, clinicians should provide instructions to patients to maximize drugs benefit/efficacy, including the fact that there is a non-linear dose-response effects across PDE5i medications, and on-demand dosing versus daily dosing for tadalafil appears to produce the same level of efficacy (*Strong Recommendation; Evidence Level: Grade C*).

**Figure 5:**
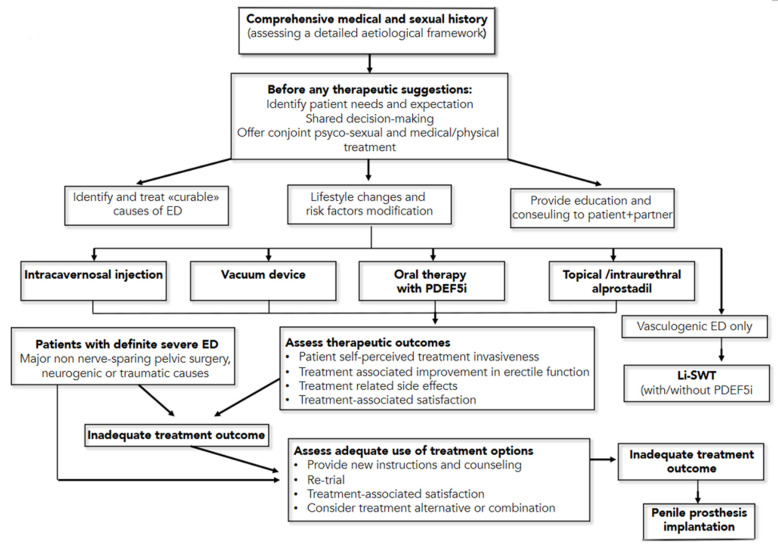
Management Algorithm for erectile dysfunction (from European Association of Urology Pocket Guidelines 2020, p. 222). ED = erectile dysfunction; PDEF5i = phosphodiesterase type 5 inhibitors; Li-SWT = low-intensity shockwave treatment.

Moreover, the dose of PDE5i should be titrated to provide optimal efficacy (Strong Recommendation; Evidence Level: Grade B). The use of nitrate-containing medications, combined with a PDE5i, can cause severe hypotension and anginal symptoms in patients affected by CAD. As such, men taking nitrates should not use PDE5i medications. Patients should also be informed regarding a vacuum device’s treatment option to treat arteriogenic erectile dysfunction (*Moderate Recommendation; Evidence Level: Grade C*). Men with ED should be informed regarding the treatment option of either intraurethral (IU) alprostadil or intracavernosal injection (ICI—with alprostadil, papaverine, phentolamine, and/or atropine) and an in-office training of both treatments options is recommendable (*Conditional Recommendation; Evidence Level: Grade C*). For men with ED, low-intensity extracorporeal shock wave therapy, platelet-rich plasma therapy and intracavernosal stem cell therapy must still be considered investigational (*Conditional Recommendation; Evidence Level: Grade C*). For young men with ED and focal pelvic/penile arterial occlusion and without documented generalized vascular disease or veno-occlusive dysfunction, penile surgical arterial reconstruction should be considered (*Conditional Recommendation, Evidence Level: Grade C*), while penile venous ligation by surgery is not (*Moderate Recommendation, Evidence Level: Grade C*). Patients should be informed regarding the treatment option of penile prosthesis implantation, including discussing benefits and risks/burdens (*Strong Recommendation, Evidence Level: Grade C*). A summary of these guidelines’ recommendation is reported in Figure 5. 

## 7. New on the Horizon: Regeneration Strategies for ED

### 7.1. Platelet-Rich Plasma and ED

Platelet-rich plasma (PRP) is autologous blood plasma that contains platelet concentrations that exceed physiological standards by 3–7-fold. When platelet concentration is >1,000,000 U/mL in the final centrifuge product, PRP shows its therapeutic effect. Since PRP contains numerous growth factors, such as vascular endothelial growth factor [73]; platelet-derived growth factor (PDGF) [74]; fibroblast growth factors [75]; epidermal growth factor [76]; and insulin-like growth factor-1 [77], its regenerative potential has been utilized in the treatment of ED for blood flow restoration, extracellular matrix composition stabilization, and endothelial regeneration.

In 2009, Ding et al. [78] studied the effect on regeneration and restoration of the cavernous nerve function after its damage in a rat model by PRP injection. Intracavernous pressure (ICP) was measured to evaluate erectile function (EF). In the PRP group both EF and ICP improved significantly compared to untreated animals (*p* ≤ 0.05). Moreover, the myelination of cavernous nerve fibers in the non-treated group was significantly lower than PRP-treated animals. An experimental study by Wu and co-authors [79] injected PRP in the intralesional site immediately after cavernous nerve damage. Four weeks later, ICP was 1/3 higher in rats treated PRP vs. untreated group of animals (*p* ≤ 0.05). At the histopathologic examination the number of myelinated axons of the cavernous and dorsal nerves was significantly higher in PRP treated animals. This finding was couple by a significant reduction of apoptotic markers and cavernous body fibrosis (prevalence of type I collagen and absence of type III collagen) in PRP treated animals (*p* ≤ 0.05). In another study, the same authors [80] optimized PRP production technology in humans, revealing that the most effective activators (chitosan, serotonin) and incubation temperature contributed to the release of a larger number of platelet-derived growth factor (PDGF). Compared to the control group, intracavernous pressure and nitric oxide synthase-levels were similar in treated animals, suggesting that optimized PRP may promote the recovery of EF. In 2013, Epifanova and associates [81] conducted an RCT dividing 75 patients into three groups: 30 patients who received intralesional therapies with PRP activated by 10% CaCl_2_ solution (Group 1); 30 patients who received intralesional therapies with PRP activated with 10% CaCl_2_ solution, combined with phosphodiesterase type 5 inhibitors ((PDE-5i—Group 2); and 15 patients who received inactivated PRP (Group 3). For three weeks all pts received an injection of PRP. EF improvement was observed from twenty-eight days since treatment and persisted throughout the entire FU period. In group 1, at the Doppler examination, a statistically significant increase in peak systolic velocity (PSV) (*p* ≤ 0.005) and resistance index (RI) (*p* ≤ 0.001), as well as in International Index of Erectile Function-5 (IIEF-5) questionnaire (*p* ≤ 0.046) and Sexual Encounter Profile (SEP) (*p* ≤ 0.001) scores, was observed. In group 2, PSV (*p* ≤ 0.028) and RI (*p* ≤ 0.129) values, as well as IIEF-5 (*p* ≤ 0.046) and SEP (*p* ≤ 0.05) scores, improved. In group 3, a statistically significant difference was found in IIEF-5 and SEP (*p* ≤ 0.05) scores, as well as in PSV and RI (*p* ≤ 0.05) values. Matz et al. [82] evaluated the safety and feasibility of platelet-rich fibrin matrix (PRFM) for the treatment of ED. Patients received 1–8 injections of 4–9 mL of PRFM (mean injections 2.1 per patient). The IIEF-5 score increased on average by 4.14 points and no adverse effects were recorded during FU period of fifteen months. 

To summarize, despite PRP is a promising therapeutic option, more quality animal studies are required to better understand PRP action’s mechanism before such therapy may be approved by regulatory authorities and move into the clinical arena [82].

### 7.2. Stem Cell Therapy and ED

Depending on the surrounding environment, stem cells are undifferentiated cells capable of differentiation in various types of cells, including endothelial cells, smooth muscle cells, and neurons. The rationale for their therapeutic use in ED is based on two principal hypotheses: the first is that their transplantation into the penis might replenish dysfunctional endothelial cells or cavernous smooth muscle cells; the second is that transplanted stem cells might act through paracrine mechanisms by inducing regeneration of the host’s own cells’ or by reactivating proper interactions between endothelial cells and cavernous SMCs [83]. Indeed, the latter seems to be the primary stem cell action in acute disease models, such as the one represented by cavernous nerve injury. However, experimental studies showed that only few stem cells could be detected after transplantation after an acute injury and there is no evidence that transplanted stem cells are able to differentia into endothelial cells, vascular smooth muscle cells, or nerves [84]. Moreover, since there is no recognizable and temporally defined acute injury in chronic erectile dysfunction models, such as diabetes mellitus and hyperlipidemia, stem cell therapy’s mechanism of action is more complex to investigate in this setting [85].

Stem cell types tested in erectile dysfunction include adipose tissue-derived stem cells (ADSCs), bone marrow-derived stem cells (BMDS), and muscle-derived stem cells [85].

In 2015, Shan et al. [84] investigated the effects of stem cell therapy in cavernous nerve injury (CNI). The authors performed a meta-analysis evaluating data from twelve published preclinical studies involving animals treated with stem cells. They found that stem cell therapy can improve EF by increasing intracavernosal pressure/mean arterial pressure ratio. Moreover, uncultured stem cells (adipose-derived stromal vascular fraction and bone marrow mononuclear cells) seem to be less effective than cultured stem cells in improving EF. Another important finding is that different species, sources, and types of stem cells elicited similar effects on rats with CNI, as did different delivery approaches (peri-prostatic, major pelvic ganglion, or intracavernosal injection). Acellular scaffolds used to locate the stem cells in the ganglia or injured cavernous nerve appear advantageous for nNOS expression (meaning cavernous nerve regeneration) but not for α-SMA (alpha smooth muscle actin) expression recovery.

Human data on stem-cell therapy for ED has also been recently reported. Bahk et al. [86] evaluated the effect of intracavernosal injections of umbilical cord stem cells in 7 men (mean age, 69.5 years; range, 57∼87 years) with type 2 DED. The patients received into the corpus cavernosum 1.5 × 107 human umbilical cord blood stem cells. For the three men in the control group, a sham procedure with normal saline was injected. Erectile function was assessed using the IIEF-5 and SEP questionnaires, the Global Assessment Questionnaire, and an erection diary at nine months, with a follow-up period of eleven months. The controls reported no changes in erectile function after injection. Six out of seven patients in the study group reported the return of morning erections two-months after treatment, which was maintained for at least three months. Six patients reported increased penile hardness, and by adding 100 mg of sildenafil, two patients were able to achieve an erection adequate for coitus. This effect was retained in the fifth month. At the nine-month follow-up, one patient reported the inability to penetrate even with the oral agent’s addition. Three of the seven patients agreed that stem-cell therapy had some effect, although it was insufficient to restore a normal EF, and five regarded stem-cell treatment effective when combined with a PDE5i. 

In 2016, Yiou et al. [87] presented the results at 1-year, of a nonrandomized dose-escalation pilot trial regarding intracavernous injection of bone marrow mononuclear cells (BM-MNC) in post-prostatectomy patients with vasculogenic ED. Twelve patients (aged 45∼70 years old; mean age, 63.6 years) with localized prostate cancer and vasculogenic ED (penile arterial insufficiency and/or venous occlusive dysfunction) refractory to maximal medical treatment were divided into four groups based on the amount of cells injected (2 × 10^7^, 2 × 10^8^, 1 × 10^9^, or 2 × 10^9^ stem cells). The primary endpoint was injection tolerance, while secondary endpoints were IEF score increase and penile vascularization at six months by Duplex. Endothelial function was also assessed using the penile nitric oxide release test, measuring the percentage of post-occlusive changes in cavernosal artery diameter. At 6 months significant improvements of IIEF-5 (mean score 17.4, 8.9, 7.3, and 4.5; *p* = 0.006) were observed in the total population compared to baseline. Patients on higher doses showed significantly greater improvement of spontaneous erections. At Duplex examination, improvements in peak systolic velocity were observed (normal in 7 out of 11 patients) and percent penile NO release test at six months (normal in 8 out of 11 patients were in the normal range. These clinical benefits were sustained after one year. 

Haahr et al. [88] reported the results of a 12-month prospective, open-label, phase I single-arm study. The authors evaluated the safety and efficacy of a single intracavernous injection of autologous ADSCs in 21 men with severe, post-prostatectomy ED. Patients received between 8.4 × 10^6^ and 37.2 × 10^6^ ADSCs immediately after cell isolation from liposuction. Eight patients reported a recovery of erectile function with the ability to accomplish normal tumescence for sexual intercourse.

Furthermore, Levy et al. [89] reported on the feasibility and efficacy of intracavernous injection of placental matrix-derived stem cells in eight patients. At six months, peak systolic velocity was significantly improved from 25.5∼56.5 cm/s at three months to 50.7∼73.9 cm/s, while changes in IIEF scores were not statistically significant. At 2-month follow-up, two patients were able to have erections using PDE5i. At a 3-month follow-up, three patients could attain erections with pharmacologic assistance from PDE5is, whereas previously they could not.

In 2012, Casiraghi et al. [90] published a review incorporating more than 700 patients treated by autologous or third-party bone marrow or adipose-tissue derived mesenchymal stem/stromal cells, evaluating stem-cell ability to trigger tumor growth from trials across various specialties (hematology, oncology, cardiology, neurology, and orthopedics). The safety data from clinical trials receiving does not suggest that serious adverse events are a clinically significant issue.

In conclusion, from experimental and initial clinical experience, stem-cells therapy may be an important therapeutic option for ED patients. However, despite the overwhelming enthusiasm, future studies are needed to define their safety profile and efficacy and establish the exact mechanism of action, the ideal timing for the injections, type of cells, and dosage.

## 8. Conclusions

Erectile dysfunction is common in CVD patients, and it is clear that it may represent a significative earlier predictor of cardiovascular events and cardiovascular death. Usually, there is a 3 to 5 years-time interval between the onset of ED and cardiovascular event. Thus, sexual function assessment should be incorporated into CVD risk stratification for all men since it is tightly associated with the same risk factors and share several common molecular and pathogenetic mechanisms related to endothelial dysfunction and atherosclerotic plaque formation. It should be considered that a comprehensive reduction of major cardiovascular risk factors improves overall vascular health, including sexual function.

## Figures and Tables

**Table 1:** Etiologies of Erectile Dysfunction (ED).

**Neurogenic** Central (cerebral or spinal cord): for example, cerebral insult, multiple sclerosis, and spinal cord injury Peripheral afferent (sensory neuropathy, i.e., diabetes mellitus and polyneuropathy of various other causes) Peripheral efferent (autonomic neuropathy or after radical pelvic surgery)
**Endocrinologic** Diabetes mellitus Hypogonadism, Hyperprolactinemia
**Vasculogenic** Arterial: macro or micro angiopathy (i.e., atherosclerosis and trauma) Venous: failure of the corporal veno-occlusive mechanism Sinusoidal: failure to relax (i.e., fibrosis)
**Drug-induced depression** Antihypertensives, antidepressants, antiandrogens, and major tranquillizers Cigarette smoking Alcoholism Recreational drug use (i.e., marijuana and heroin)
**Systemic diseases and general ill health** Liver disease Renal disease Respiratory disease Cardiovascular disease
**Local penile (cavernous) factors** Cavernous fibrosis after priapism or due to other reasons Peyronie’s disease Penile fracture

## Data Availability

Not applicable.
